# Volatile Organic Compounds (VOCs) as Environmental Pollutants: Occurrence and Mitigation Using Nanomaterials

**DOI:** 10.3390/ijerph182413147

**Published:** 2021-12-13

**Authors:** Elena David, Violeta-Carolina Niculescu

**Affiliations:** National Research and Development Institute for Cryogenic and Isotopic Technologies—ICSI Ramnicu Valcea, 240050 Ramnicu Valcea, Romania; Elena.David@icsi.ro

**Keywords:** environment, nanomaterial, pollution, VOC

## Abstract

Volatile organic compounds (VOCs) comprise various organic chemicals which are released as gases from different liquids or solids. The nature and impact of the health effects are dependent on the VOCs concentrations and, also, on the exposure time. VOCs are present in different household, industrial or commercial and products, but their accumulation in air and water has primarily gained attention. Among VOCs, trichloroethylene and vinyl chloride are the most toxic and carcinogenic compounds. In order to improve the indoor air and water quality, VOCs can be removed via efficient approaches involving nanomaterials, by using techniques such as adsorption, catalysis or photocatalysis. In the recent years, the development of manufacturing procedures, characterization techniques and testing processes has resulted in the growth of na-nomaterials obtaining and applications, creating great possibilities and also a tremendous prov-ocation in applying them for highly efficient VOCs removal. This review is intended to contrib-ute to the improvement of awareness and knowledge on the great potential that nanomaterials have in VOCs removal, in order a to improve indoor and outdoor environment, but also the worldwide water sources.

## 1. Introduction

Volatile organic compounds (VOCs) are organic chemical compounds found in various products that easily vaporise and reach in the environment under normal conditions. VOCs have increased volatility, mobility and they are resistant to degradation, being able to be transported to long distances in the environment [1]. The most common VOCs are the aromatic hydrocarbons, such as benzene, toluene, xylene and ethyl benzene, and halogenated hydrocarbons, such as chloroethylene and trichloroethylene. A distinguished set of VOCs are the cancerous volatile organic compounds (cVOCs), being able to cause cancer in human beings.

The most common exposure pathways to VOCs from contaminated waters were identified as drinking, bathing, food, swimming or laundries [2].

**Figure 1:**
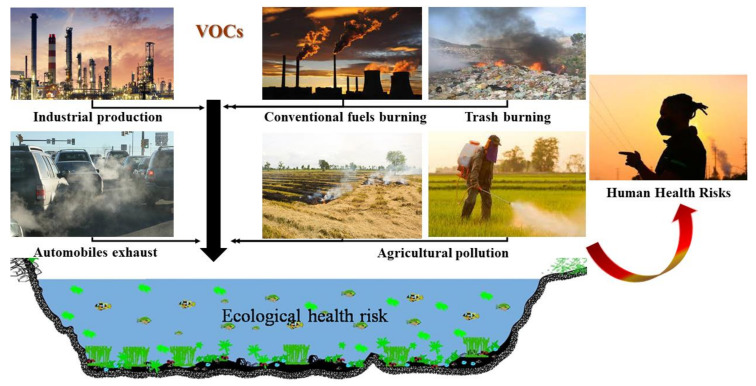
Potential sources of VOC occurrence.

The sources of VOCs are both natural and anthropogenic. Natural sources comprise emissions from plants, forest fires occurring from natural causes and anaerobic moors processes. VOCs generated by anthropogenic processes are both domestic and industrial processes: food extraction, fertilizers and pesticides use, septic system, chlorination, traffic, hydrocarbon fuels burning, petroleum storage and distribution, textile cleaning, printing, pharmaceutical industries, etc (Figure 1) [3].

The increase of worldwide agriculture activities (the use of fuels, agricultural waste burning and VOCs use as inert ingredients in pesticides) resulted in VOCs extensive distribution in the environment [4,5]. Additionally, many studies reported VOCs as indoor air pollutants, the pollution sources being tobacco smoke, chlorinated water, the use of perfumes, paint removers, adhesives, new clothing, plastics or kerosene heaters [6]. Some researchers found VOCs in bottled water [7], while others reported microbial volatile organic compounds (mVOC) in the air, probably generated from airborne microbial metabolites or spores of fungus [8]. A major source of VOCs was identified as composting [9]. Solvents that contain ionic liquids can also produce VOCs [10]. Up to now, USEPA was able to classify about 189 air pollutants, 97 being VOCs [11].

Certain VOCs were identified as greenhouse gases [12], which are able to absorb radiated energy from Earth, their concentrations increase in the atmosphere been correlated with the global warming [13]. Furthermore, VOCs from wastewaters decrease the possibility of water reuse, such as in irrigation, thereby placing a higher demand on the limited existing primary water resources [5].

One of the most important users of organic solvents (such as ethanol, isopropyl alcohol toluene or xylene) is the pharmaceutical, significant emissions of VOCs resulting from chemical synthesis or extraction steps industry [14].

Efficient technologies are necessary to reduce VOCs concentrations, by carefully designing the materials that are able to chelate, adsorb or chemically modify them, while also considering the efficacity, reuse and costs of the obtaining. Conventional methods for VOCs mitigation involve incineration [15], biological oxidation [16], chemical oxidation [15] or the adsorption on various carbon materials [17]. One of the latest trends in VOCs mitigation is the use of nanomaterials, in an effort to decrease environmental pollution [18].

In this review we focused on briefly classifying VOCs and the use of nano-scaled materials for VOCs mitigation.

## 2. VOCs Classification

Organic chemicals known as volatile organic compounds (VOCs) adversely affect the environment and human health. They evaporate at room temperature and normal pressure and can be present in both closed and open spaces. Outdoor VOCs affect the natural environment and indirectly affect human health, while their presence indoors can affect human health. Some of the volatile organic compounds are more volatile than others, those that evaporate faster are more dangerous and pose a higher risk to the environment and humans. Organic pollutants in terms of volatility are classified into three groups [19,20,21,22]. This classification is important to indoor air and is considered as definition of indoor volatile organic compounds [23,24,25]. The main sources of VOCs are considered to be the following [26,27,28]:

(i) Exploitation and use of fossil fuels, e.g., incomplete burning of fossil fuels or their evaporation;
(ii) Solvents used in paints and inks; approximately 12 billion litres of paints are produced annually and the usual solvents are aliphatic hydrocarbons, ethyl acetate, glycol ethers, acetone, etc. [27];
(iii) The resulting products in the form of compressed aerosols, mainly butane and propane, contribute globally to 1.3 billion tonnes of VOC emissions per year [19];
(iv) Use of biofuels, for example, cooking oils, bioethanol, bio-fuels;
(v) Biomass combustion, especially from forests and agricultural wastes, although in principle the combustion results in carbon dioxide and water, incomplete combustion leads to a variety of VOCs;
(vi) Toxic volatile organic compound released from metalworking fluids (MWFs);
(vii) Incineration of household wastes and other sources.

Table 1 presents the general classification of VOCs, as a function of their volatile characteristics.

***Very Volatile Organic Compounds (VVOCs)*** represent the most dangerous pollutants class and they are toxic at very low concentrations [19,22,25,28]. This class includes propane, butane, methyl chloride, etc. Propane (C_3_H_8_) is highly dangerous, it is shipped as a liquefied gas under its vapour pressure and is used for heating or cooking. Butane (C_4_H_10_) is used also similar to propane and it is regarded as one of the more harmful volatile compounds to inhale. Methyl chloride (CH_3_Cl) known as chloromethane, is a colourless, flammable and toxic gas. It is used as a refrigerant but it has many other applications, such as solvent in petroleum refining, chlorinating and methylating agent in organic synthesis, herbicide, propellant in polystyrene foam production. The exposure to methyl chloride causes a variety of problems from drowsiness and dizziness to seizures and comas depending on the level of concentration and time of exposure. Chloroform, or trichloromethane (CHCl_3_) is a dense volatile organic compound, colourless liquid with strong-smelling that is produced on a large scale as a precursor to PTFE and to various refrigerants. It is a powerful anaesthetic and sedative when inhaled or ingested. Chloroform has induced liver tumours and kidney tumours in mice and rats and the hepatotoxicity and nephrotoxicity of chloroform is considered to be due to phosgene.

***Volatile organic compounds (VOCs)*** are no less dangerous than VVOC_S_, are found in household products and can be present in environment [19,22,25,28]. These include formaldehyde, vinyl chloride, carbon tetrachloride, toluene, acetone, isopropyl alcohol, hexanal, carbon disulfide, etc.

*Formaldehyde* (CH_2_O) is used in making of resins for building materials, paper, coatings for clothing fabrics, is known as a carcinogen VOC. It is commonly found in glues, cast plastics, varnishes, insulating materials, pressed wood products such as plywood, particle board, laminate flooring.

*Vinyl chloride* (C_2_H_3_Cl) is used in making of plastics (PVC), floor coverings and consumer goods and is also known as chloroethene, chloroethylene or ethylene monochloride. It is “highly likely to be carcinogenic” and the people residing close to factories that produce vinyl chloride are exposed at risk. The liver is mainly affected by the toxicity of vinyl chloride, by exposure to air containing vapours of vinyl chloride, liver damage occurs, and liver function is affected.

*Trichloroethylene* is a halocarbon mainly used as an industrial solvent. It is a clear liquid, colourless non-flammable, similar to chloroform, having a sweet smell. Groundwater and drinking water contamination are due to industrial discharge, being a major concern for human health and over time causing numerous incidents and lawsuits. The exposure to trichloroethylene is mainly due to drinking water consumption. Higher concentrations of trichloroethylene result in tachypnoea. Additionally, many types of cardiac arrhythmias could occur, and they are accented by epinephrine (adrenaline). The symptoms of the acute non-medical exposure to trichloroethylene are similar to those of an alcohol intoxication, beginning with headache and dizziness, confusion and progressing with the increasing exposure to state of unconsciousness. Respiratory and circulatory depression could result in death.

*Carbon tetrachloride* (CCl4) is an organic compound with the chemical formula CCl4, a colourless liquid with a sweet smell that can be detected at low levels. It is not flammable at lower temperatures, and it is used in fire extinguishers, as a precursor agent to refrigerants and also as a cleaning agent, but due to environmental issues it is less and less used. After exposure to high concentrations of carbon tetrachloride (especially vapor), the central nervous system could be affected and degenerate the liver and kidneys, long time exposure could be fatal.

*Toluene* (C_7_H_8_) is an aromatic hydrocarbon, colourless liquid, water-insoluble with the smell associated with paint thinners and it is predominantly used as an industrial feedstock and a solvent in some types of paint thinner, permanent markers, some types of glue. Toluene has the potential of causing severe neurological harm.

*Acetone* is an organic compound with the formula (CH_3_)_2_CO, is the simplest and smallest ketone. It is a colourless liquid, highly volatile and flammable with a characteristic pungent odour, miscible with water and it is used as an important organic solvent. It is also used for production of methacrylate, as well as bisphenol A and in organic chemistry it is a common building block. It is a volatile organic compound (VOC) and could affect human body, the people with diabetic problems are mainly affected.

*Isopropyl alcohol* is a colourless liquid, flammable chemical compound, with the chemical formula CH_3_CHOHCH_3_ and with a strong odour. It is used in making of cosmetics, pharmaceuticals, perfumes, dye solutions, antifreezes, soaps, cleaner and disinfecting agent. Prolonged exposure causes respiratory problems.

*Hexanal or hexaldehyde* (C_6_H_12_O) is used as a flavouring in food industry and as a fragrance in perfumes or to obtain other chemicals that are used in the making of plastics, rubbers and insecticides. People exposed to moderate concentrations of hexanal for no long time can suffer irritation of the nose, throat, lungs, eyes and skin. A longer periods or higher exposure determine a choking feeling, coughing and rapid breathing.

*Carbon disulfide or carbon bisulfide* (CS_2_) is a highly volatile compound and is used in the manufacturing of viscose rayon and cellophane. It is also present as compound in varnishes, solvents and insecticides. By inhalation in an occupational setting arrives into human body and can cause respiratory problems.

***Semi volatile organic compounds(SVOCs)*** are substances with a higher molecular weight and boiling point than VOCs and it is less likely to become vapours at room temperature, but this does not mean they are any less dangerous to people and environment [19,22,25,28]. The use of SVOCs in building materials, furnishings, electronics, and furniture as additives can produce serious problems due to toxicity. Some examples of such products are presented below.

*Pesticides*, especially organochlorine pesticides, are extensively used in agriculture. As neurotoxicants they caused severe health and environmental problems.

*Chlordane* (C_10_H_6_Cl_8_), is considered as a possible human carcinogen compound, is an organochlorine compound used as a pesticide. It is a white solid and as with other chlorinated cyclodiene insecticides, it is classified as an organic pollutant hazardous for human health and a pollutant for environment. Chlordane is resistant to degradation in the environment and in humans/animals bodies and readily accumulates in lipids (fats) and the exposure to this compound has been linked to cancers, diabetes, and neurological disorders.

*Benzyl alcohol* (C_7_H_8_O), is used as a solvent, a preservative, to manufacture chemicals, as a fragrance in perfumes and flavouring, and also as an ingredient in cosmetics. Benzyl alcohol is also used in inks, as a photographic developer, and in dyeing nylon filament, textiles and sheet plastics. Exposure to very high concentrations could result in toxic effects such as respiratory failure, vasodilation, hypotension, convulsions, and paralysis.

*Fire retardants* represent a significant source of SVOCs and they are found in fire extinguishers. Polychlorinated biphenyls (PCBs or PBBs) are the most common SVOCs. A fire retardant is a chemical substance that is usually used to slow down or stop the spread of fire or to reduce its intensity. This processes are commonly accomplished by chemical reactions that can reduce the flammability of fuels or delay their combustion and can cool the fuel through physical action or endothermic chemical reactions. Fire retardants are generally considered non-toxic, but even less-toxic compounds carry some risk when organisms are exposed to large amounts because they are releasing dioxins and furans during combustion.

Among *plasticizers*, phthalates, polybrominated diphenyl ethers or hexabromocyclododecanes are often used in construction and interior design materials (soft furniture, flooring, wallpaper, paints), cosmetics, toys or personal care products. Phthalates can induce in children asthma, allergies, neuropsychiatric effects or body weight gain [29]. Polybrominated diphenyl ethers or hexabromocyclododecanes negatively impact children’s endocrine, neurologic or reproductive systems.

## 3. VOCs Impact

VOCs have a variety of direct and indirect impacts on people and the environment and the main problems refer to: harmful effects on people health and on environment through toxicity; carcinogenicity and other adverse effects; the damage to materials; the tropospheric photochemical oxidant formation; stratospheric ozone depletion; global climate change; odour released.

Many VOCs can cause damage to materials near their point of discharge, as a consequence of oxidizing or corrosive properties. VOCs can indirectly contribute to material damage by the formation of ozone which is a very strong oxidizing agent and can attack materials such as natural and synthetic rubber, textiles and resins, or those used in surface coatings. Accelerated degradation of buildings occurs through damage to the protective layers. On the other hand, between VOCs and nitrogen oxides (NOx) reactions occur in the presence of sunlight and result in photochemical oxidants (including ozone, peroxyacyl nitrates, peroxides, etc.) [28]. These chemicals can affect human health and are harmful to the environment, increasing harmfulness of NO to the environment by its oxidation to NO_2_.

Almost all VOCs directly contribute to global warming by absorbing infrared radiation from the earth’s surface, and the more complex a VOC is, the greater its ability to absorb infrared radiation, yet most VOCs have a short atmospheric lifetime and are decomposed, thus diminishing their effect (the exceptions to this rule are saturated light hydrocarbons and halogenated compounds). VOCs indirectly contribute to global warming by changing the concentration of ozone, which is a strong greenhouse gas [22].

It has been demonstrated that organic vapours are the cause for the majority of particle growth in various media having a source of organic precursor gases, however, their complexity determined a challenge with respect of their detection and contribution to nanoparticle growth [30]. One can simply deduce that the smaller the particles, the volatility of a compound has to be the lower so it can be able to condense [30]. Oxidation of VOCs results in abundant organic acids, which are supposed to conduct aerosol nucleation [31]. Furthermore, photooxidation of vehicular exhaust (containing aromatic VOCs) results in the presence of abundant precursors for nucleation and growth of ultrafine particles in the air, as it was previously demonstrated [31].

Many VOCs have a characteristic odour and, in some situations, can occur with VOC emissions and unpleasant odour problems [26]. The odour intensity of a particular compound is usually expressed by its odour threshold, i.e., the concentration at which half the population could not detect that odour. It is difficult to predict the odour threshold of a VOC mixture, as there are often complex and nonlinear synergistic effects that can alter both the intensity and quality of the perceived odour and in such situations, the odour threshold emitted by the VOC mixture must be established by practical measurement.

VOCs are ubiquitous in indoor air; the questions are about what concentration levels are allowed in the air and how long the people can be exposed to them without risk. Using sensors to determine air quality and measure VOC concentrations is a way to detect elevated levels and avoid exposure to these compounds.

## 4. VOCs Mitigation by Nanomaterials Use

Various nanomaterials have been developed for VOCs mitigation, but their elimination mechanisms depend on the nanomaterial’s physicochemical characteristics such as porosity, size, electrostatic interaction, surface functionality or chemical composition. Various studies reported the efficient application of nanomaterials for VOCs mitigation, in an effort to decrease environmental pollution [18]. Such materials include, but are not limited to, carbon nanomaterials [32], metallic and metal oxide nanomaterials [33,34] or polymer nanocomposites [35,36]. Several investigations reported the use of nano- and micro-scale materials for the adsorption of VOCs from atmosphere and water [15].

The BTEX VOCs (benzene-B, toluene-T, ethylbenzene-E and p-xylene-X) are solvents intensively used in various branches of the industry, high volumes of BTEX wastewater being discharged into environment, causing environmental risks and threatening public health [37]. Various nanomaterials, such as carbon nanotubes (CNTs) were used for the mitigation of BTEX from contaminated water [37].

For example, multiwall carbon nanotubes (MWCNTs) obtained by catalytic chemical vapour deposition and oxidation with sodium hypochlorite (NaOCl), were applied for adsorption of BTEX from water [37]. The affinity of BTEX toward the prepared carbon nanotubes followed the order: X > E > T > B. this may be explained by the interaction of various factors, such as the molecular weight variation (B < T < E, X), the solubility decrease (B > T > E > X) and the increase in boiling point (B < T < E, X).

For comparison, various CNTs oxidized by other chemical agents 9 such as HCl, HNO_3_, H_2_SO_4_ or NaOCl) were used for BTEX adsorption from aqueous solutions [38]. The results highlighted that NaOCl-oxidized MWCNTs showed the greatest adsorption, followed by HNO_3_- and H_2_SO_4_-oxidized carbon nanotubes. The mechanism of the adsorption was associated with a π–π electron–donor–acceptor mechanism, in which the carboxylic oxygen atom of the MWCNTs acted as the electron donor and the BTEX aromatic ring acted as the electron acceptor, similar to the BTEX adsorption mechanism on powdered activated carbon [38].

MWCNTs were also applied as solid-phase extraction (SPE) sorbents for chlorobenzenes removal and compared the adsorption with SPE adsorbents such as activated carbon or C18 silica [39]. The adsorption capacities of MWCNTs were similar with the ones of the classical adsorbents, showing that MWCNTs can be efficiently used both for chlorobenzenes and other VOCs determination from natural or polluted waters. Carbon nanotubes were used for 1,2-dichlorobenzene adsorption from aqueous solutions, a maximum sorption capacity of 30.8 mg/g being reached in 40 min [40].

The final goal of nanomaterials synthesis is to obtain an efficient adsorbent for the selective mitigation of VOCs with different functionalities. Accordingly, periodic mesoporous organosilica nanoparticles (MO SiNPs) were synthesized by an efficient one-pot condensation process, the resulted nanomaterials (with approximately 400 nm diameter) having high surface areas (977 m^2^/g) and large pore volume (0.92 cm^3^/g) [15]. They were applied for the capture of hexanal and butyric acid vapours using a GC capture assay, the PMO SiNPs exhibiting a mitigation efficiency higher than 99% for both VOCs even at low adsorbent dose. The study showed also that the nanomaterials can be reused for several cycles [15].

An organic–inorganic hydrophobic mesoporous silica has been successfully obtained by a co-condensation method with tetraethoxysilane (TEOS) and vinyltriethoxysilane (VTES) under acidic condition [41]. This nanomaterial, denoted as v-SiO_2_ has been used for the adsorption of p-xylene. Adsorption tests demonstrated that the hydrophobicity of the nanomaterial against p-xylene adsorption (85.2%) is significantly improved toward pure silica (56.2%) [41].

In recent years, noble metal-based nanostructures have attracted great interest for VOC catalytic oxidation, due to their high efficiency. Table 2 summarize some of the latest studies in which noble metals were used for obtaining nan- catalysts for VOCs mitigation by oxidation [42].

In order to enhance their use, the noble metals dispersion on the substrates surface must be improved. In order to activate oxygen for the VOC oxidation by noble metal catalysts supported on conventional substrates (SiO_2_, Al_2_O_3_ and TiO_2_), the electronic structure or chemical state of the metal must be changed by modifying the particle size, morphology or structure. For noble metal catalysts supported on transition-metal oxides, the activation of oxygen can be reached by growing the adsorbed oxygen quantity and enhancing the lattice oxygen mobility by doping the substrates with other transition metals [42].

Among noble-based nano-catalysts, Pt-based nanomaterials had attracted attention as catalysts for VOCs (benzene, toluene, xylene, formaldehyde and methane), being able to be easily dispersed on porous substrates. For example, mesoporous AlOOH nanoflakes on which Pt was deposited [50]. The Pt/AlOOH catalysts (0.8 wt.% Pt) presented the highest efficiency for the decomposition of formaldehyde at room temperature, the catalytic activity being determined by the high dispersion of Pt nanoparticles, abundance of surface hydroxyls groups, high adsorption property of the substrates, as well as by the high specific surface area and large pore volume [50].

Porous SBA-15 silica with high specific surface was used for Pt loading by impregnation or deposition-reduction, the resulted catalysts being applied for the benzene oxidation [51]. One of the parameters that influence the physicochemical properties and catalytic performances of Pt/SBA-15 catalysts is the synthesis method: the catalysts obtained by the reduction with NaBH_4_ and H_2_ had higher efficiency than the ones obtained by sodium citrate reduction, due to the higher dispersion, smaller crystallite size, but also to the negatively charged surface resulted from the strong metal-support interaction [51].

The VOCs catalytic oxidation is highly dependent on the size of Pt particle. For example, some researchers prepared Pt-d/ZSM-5 nano-catalysts (d is the mean diameter of the Pt nanoparticles) by loadings 1 wt.% Pt nanoparticles (with diameters ranging from 1.3 to 2.3 nm) on the surface of ZSM-5 substrate; the catalysts were tested for toluene oxidation [44]. It was observed that Pt-1.9/ZSM-5 had the highest efficiency due to Pt dispersion, which leads to a toluene conversion of 98%. Some 1 wt.% Pt/TiO_2_ having different Pt particle sizes (between 1.54 and 22.3 nm) nano-catalysts were prepared using a reduction process with NaBH_4_ and thermal treatment; they were tested in the catalytic oxidation of gaseous formaldehyde at ambient temperature [52].

Pd noble metal has relatively lower activity comparing with Pt in catalytic oxidation of most pollutants, but it is used as an active ingredient in various catalysts due to its high catalytic efficiency in the oxidation of toluene [53] or halocarbons [54]. Developing Pd-based catalysts (such as Pd-TiO) for VOCs mitigation is gaining considerable interest [46].

Au was initially considered to be a poor catalyst due to its chemical inertness molecules such as oxygen or hydrogen. However, Au nanoparticles possessing unpredictable and unique catalytic features have been intensively studied for VOC mitigation [47]. For example, the propene oxidation efficiency has been investigated using Au supported catalysts on TiO_2_ mesoporous oxide having specific surface area of about 50 m^2^/g and pore diameter between 10 and 30 nm. Au/TiO_2_ with high dispersed Au^0^ nanoparticles which strongly interacted with the mesoporous oxide, having high activity in the oxidation of propene. Additionally, the Au^+^ species found on the interface with the titania positively influenced the catalytic activity [47].

Ag also attracts great interest in the field of VOCs mitigation, especially for the catalytic oxidation of formaldehyde, even though its activity is lower than Pt, Pd or Au. Various Ag-based catalysts loaded on different supports (TiO_2_ or CeO_2_) were obtained by impregnation and they were tested for the oxidation of formaldehyde, resulting the complete formaldehyde conversion at about 95 °C [48].

To improve indoor air quality, catalytic nanomaterials can be used for VOCs reduction from coatings, paints, air filters, building materials, etc. Due to their characteristics such as non-toxic, high chemical stability, low cost, metal oxides such as TiO_2_ and ZnO have been widely investigated and dominate the field of VOCs removal application in buildings. Other nanocatalysts have shown high performance and have been theoretically and lab studied, being considered potential candidates for applications in future environmentally friendly and healthy buildings. Their development will contribute to the additional knowledge and potential in the application of catalytic nanomaterials for VOCs reducing or total removal, so that buildings will be healthier and will contribute to a better indoor and outdoor environment.

Photocatalysis based on TiO_2_ or TiO_2_-supported metal catalysts is also intensively studied as a green environmental remediation technique for VOCs removal. The photocatalyst are mainly influenced by the structure and morphology [55] or by the treatments applied on TiO2 particles [56]. The influence of synthesis parameters variation on the size and morphology of the TiO_2_ nanoparticles was studied; the trichloroethylene degradation over TiO_2_ catalyst reached a maximum value at particle size of 7 nm [55]. The photocatalytic efficiency of TiO_2_ nanotubes and TiO_2_ nanoparticles on degradation of gaseous toluene and acetaldehyde was also investigated [57]. The photocatalytic activity of TiO_2_ nanotubes was slightly reduced after the five cycles in toluene degradation, compared with TiO_2_ nanoparticles which rapid deactivated as after the cycles were repeated. The explanation resides in the highly ordered open channel structure, TiO_2_ nanotubes being able to rapidly provide O_2_ molecules to the active sites, preventing the accumulation of carbonaceous residues on the nanotubes surface [57]. It can be concluded that the structural features of the nanotubes prevent the catalyst deactivation during the photocatalytic removal of the aromatic compounds.

The photocatalytic performance of nanotubes containing TiO_2_ (TNT) was compared with the one of TiO_2_ nanoparticles (TNP) film during the repeated cycles of photocatalytic degradation of gaseous toluene and acetaldehyde, resulting that the nanotubes were more efficient for the selected VOCs removal [57].

Novel nanostructured gas filtering systems based on TiO_2_ thin films were developed using atomic layer deposition (ALD) for VOCs removal, showing higher efficiency for the adsorption of toluene [58].

Freestanding doubly open-ended TiO_2_ nanotubes (DNT) film were also synthesized, showing an improved performance and durability for the photocatalytic degradation of acetaldehyde and toluene in gaseous phase than classical TiO_2_ nanotubes [59]. Then, after the freestanding DNT film was loaded with TiO_2_ nanoparticles (NP@DNT) in the inner wall, the performance for VOCs degradation increased by 1.3 and 1.8 times compared to the ones of bare DNT and TNT, respectively. However, the loading of TiO_2_ nanoparticles on TiO_2_ nanotubes presented a lower performance than bare TNT.

As an alternative to TiO_2_, zinc oxide is a fast and efficient chemical decontamination nanomaterial used for VOCs mitigation [60]. Three synthesis methods for preparing ZnAl_2_O_4_ (solvothermal, citrate precursor and hydrothermal methods) were compared for the photocatalytic degradation of toluene in gaseous phase [61]. The performances of the ZnAl_2_O_4_ samples prepared by the solvothermal method showed about 90% photocatalytic performance for toluene removal. The photocatalytic oxidation of gaseous pollutant over UV-illuminated ZnAl_2_O_4_ proved to be a promising technique for air cleaning.

Cryptomelane-type octahedral molecular sieve (OMS-2) manganese oxide, amorphous manganese oxide (AMO) and mixed copper manganese oxide (CuO/Mn_2_O_3_) nanomaterials were prepared and compared with commercial MnO_2_ [62]. Due to structure, hydrophobicity, morphology, as well as the redox properties, OMS-2, AMO and CuO/Mn_2_O_3_ exhibited higher oxidation activities than the commercial MnO_2_. Furthermore, a novel composite catalyst was developed for long-lifetime removal of formaldehyde, by loading manganese oxide (MnO_x_) catalysts on a polyacrylonitrile-based activated carbon nanofiber (PAN-ACNF) support [63]. The combination of MnO_x_ with PAN-ACNF created synergic effects on the performance of formaldehyde removal, which improved the activity of PAN-ACNF, both in dry and humid conditions without UV light.

Other oxides were also studied, for example iridium oxide particles supported on SiO_2_ and they were used for the total oxidation of VOCs [64]. The obtained results showed that the catalytic activity increased when the size of iridium particles decreased.

Hybrid nanomaterials such as Pt-rGO-TiO_2_ were also obtained and tested for VOCs photocatalytic removal [65]. The light intensity influenced the catalytic efficiency of Pt-rGO-TiO_2_ over the toluene conversion and the resulted CO_2_ yield. At an infrared irradiation intensity of 116 mW/cm^2^, the maximum toluene conversion efficiency was 95% and the CO_2_ yield was 72% [65].

Graphene and graphene oxide (GO) have attracted much interest as a proficient matrix for gaseous pollutants adsorption, due to their characteristics, including obtaining, high surface area, pores structure and size, high chemical stability and thermal stability [66]. Nanocomposites of TiO_2_/graphene were obtained through a facile hydrothermal reaction of graphene oxide and TiO_2_ in a mixture of ethanol and water, the nano-catalyst presenting higher photocatalytic stability and efficiency than TiO_2_ in gas-phase degradation of benzene [67]. The activities of three graphene-based co-catalysts (graphene oxide, reduced graphene oxide, and few-layer graphene) were tested on gas-phase photocatalytic oxidation of methanol, the reduced graphene oxide having the highest performance with the best rate of conversion [68].

The composite of graphene oxide with metal organic frameworks such as MOF-5 was obtained and prove to be efficient in the removal of gaseous benzene, exhibiting a mitigation capacity of 251 mg/g [69]. The graphene oxide/MOF-5 nanomaterial was prepared with different quantities of graphene oxide (between 1.75 wt% and 7.0 wt%), the one prepared with 5.25 wt% having the best activity in terms of benzene removal. Additionally, the use of 5.25% GO with MOF-5 increased the surface area of MOF-5 up to 727 m^2^/g, the pores volume being also increased up to 0.35 cm^3^/g.

The graphene oxide and reduced graphene oxide had also been studied for the toluene removal [70]. The possibility of these nanomaterials to possess π–π bonds, hydrophobic and electrostatic interactions with toluene can be helpful to the toluene adsorption on their surface. Recent studies reported the synthesis of three types of graphene nanomaterials (graphene platelets-GP, rGOMW and KOH activated rGOMW (rGOMW-KOH) and their use in toluene adsorption, their efficiency being compared with the one of active carbons [71]. The graphene nanomaterials achieved toluene removal in the order rGOMW-KOH (14.4 mg/g) > rGOMW (7.0 mg/g) > GP (2.0 mg/g). It was observed that the specific surface area of the nanomaterials also had the same trend. It was supposed that the major reason for toluene adsorption was the appearance of π-π interactions (between π-electron rich region of graphene materials and toluene aromatic ring of toluene), but also the specific surface area influenced the adsorption. GP had the maximum selectivity towards toluene, its higher graphitic character being responsible for the increased adsorption to specific surface area ratio. However, rGOMWKOH presented the maximum adsorption capacity for toluene, probably due to its higher specific surface area, having similar adsorption efficiency with active carbons in terms of toluene removal [71].

Ethanol (an alcoholic VOC) removal by adsorption on graphene materials was investigated. These nanomaterials were applied as composites with MOFs (metal organic frameworks) [69], the high specific surface, porous structure and availability of oxygen functionality resulting in 158.2 mg/g adsorption capacity for ethanol. Other study reported an adsorption capacity of 635 mg/g for ethanol by using a Cu-BTC/graphene oxide composite at ambient temperature [72].

**Figure 2:**
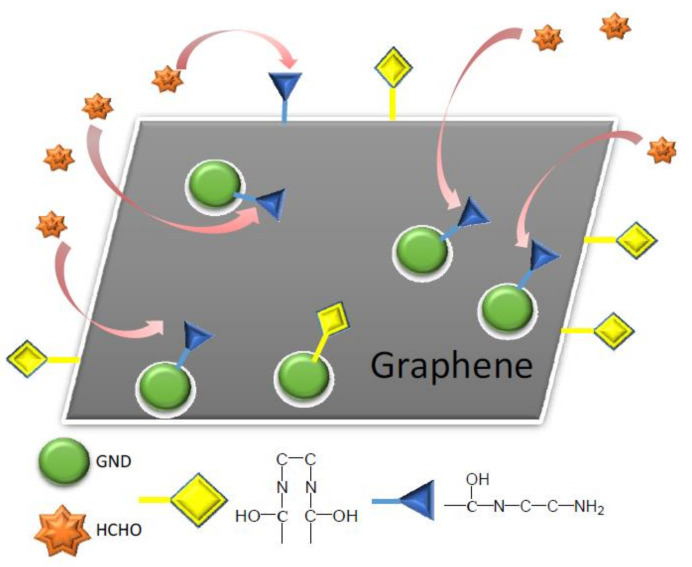
Amino graphene nanodots decorated functionalized graphene sponge—interaction with formaldehydes [73].

Graphene nanomaterials had also been used for the carbonyl VOCs removal. Various graphene materials (such as amino functionalized graphene sponge—G/S or amino functionalized graphene sponge decorated with graphene nanodots—G-GND/S) were employed to remove the indoor formaldehyde [73]. The second material was characterized by high concentration of amine groups on the surface comparing with the first one, resulting in its high interaction with the formaldehyde (Figure 2).

As a consequence, its adsorption capacity was higher (about 23 mg/g) compared to the one of the first material (about 7 mg/g).

**Figure 3:**
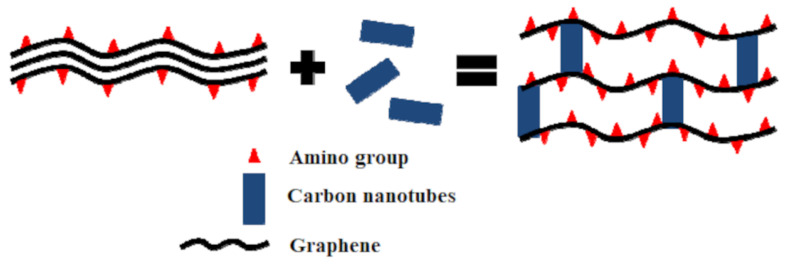
The potential mechanism of carbon nanotubes-enhanced graphene aerogel for formaldehyde removal [74].

Other studies reported the use of amino-functionalized graphene aerogel as a composite with carbon nanotubes in order to remove gaseous formaldehyde [74]. The adsorption of formaldehyde was both chemical and physical. The chemical adsorption process was due to the van der Waals forces between the amino groups of graphene and the carbonyl group from formaldehyde. In the case of the composite material, carbon nanotubes induced the reduction of pore diameters, resulting the exposure of more amino groups for the VOC uptake (Figure 3), reaching a maximum adsorption capacity of about 27 mg/g.

Graphene nanomaterials were also tested for the removal of chlorinated VOCs, such as methylene chloride or carbon tetrachloride. Graphene oxide combined with MOFs such as MIL-101 (Cr) enhanced the carbon tetrachloride adsorption capacity up to 2368 mg/g, several parameters being responsible for the composite efficiency such as high specific surface area, increased dispersive forces, the ability to generate more defects and the formation of new pores) [75].

Summarizing, the use of graphene nanomaterials highlighted great potential for various VOCs mitigation. However, these nanomaterials can be underperforming in practice, where the concentration of the pollutants is not as high as in laboratory experiments. To avoid such milestone, it is important to assess the nanomaterials efficiency by reducing the sources of bias.

## 5. Conclusions

The detection and analysis of VOCs from environment is a major provocation due to the issues raised from their sampling and actual analysis. These VOCs can be theoretically removed, among other classical techniques, by adsorption, catalysis or photocatalysis.

The regeneration and reusability of any adsorbent or catalyst are crucial parameters in evaluating the operational costs and viability for industrial uses. Catalytic and photocatalytic nanomaterials are gaining interest for VOCs mitigation. Among them, up to now, TiO_2_ is the most common, efficient and economical due to the low cost, high chemical stability, and low toxicity, being applied in the catalytic VOCs removal from buildings. As the photocatalytic efficiency of the metal oxides can be improved by combining with hybrid adsorbents, more catalysts nanomaterials are to be developed. In order to improve the removal of the indoor VOCs, more investigations should be achieved to grow the efficiency in particular conditions such as visible light. One of the greatest issues is the deactivation of catalysts under real process operation. In order to surpass this milestone, a mandatory step in catalysts preparation must be their aging under harsh pre-treatment, in order to insure their durability and stability.

The understanding of the catalytic mechanisms, for example, the interface boundary sites and the synergetic effect, may be helpful for the development of highly efficacy and stable catalysts with interesting design and tailored functionalities. Up to now, the mechanisms for the catalytic oxidation of VOCs with small molecules have been intensively studied and most of the intermediates were identified. However, the mechanisms for catalytic oxidation of VOCs with large molecules are still under investigation due to the complicated reaction pathways. The elucidation of the reaction mechanisms will also stimulate the developing of new and improved characterization techniques, especially for in situ analysis. Furthermore, up to this moment, only few studies were performed for the catalytic oxidation of mixed VOCs from actual industrial processes and from indoor environments, the performance and mechanism being quite different from those performed in laboratory conditions using single VOC. Therefore, more academic and industrial research must be performed in order to be able to extensively apply nanomaterials for VOCs mitigation.

This investigation tried to highlight the fundamentals of VOCs mitigation by using nanomaterials, so that it can be helpful for the establishment of more efficient technical approaches for the pollutant’s removal from the environment.

Screening the population for health risks must be periodically achieved, strengthening the awareness about the safe practices for waste disposal and indoor air pollution being able to minimize the risk for unintentional or intentional VOCs contamination.

## Figures and Tables

**Table 1:** Classification of VOCs pollutants.

Class	Examples of Compounds	Boiling Point Range °C
Very volatile organic compounds (VVOCs)	propane, butane, methyl-chloride	0 to 50–100
Volatile organic compounds (VOCs)	formaldehyde, toluene, acetone, isopropyl alcohol	50–100 to 240–260
Semi volatile organic compounds (SVOCs)	pesticides (chlordane, DDT), plasticizers (phthalates)	240–260 to 380–400

**Table 2:** Noble metal-based nano-catalysts for VOCs oxidation.

Catalyst	Preparation Method	Loading (wt%)	Catalyst Mass (mg)	VOC Type	VOC Concentration (ppm)	Reference
Pt/TiO_2_	impregnation	0.01–1.00	250	formaldehyde	22	[43]
Pt/ZSM-5	reduction	0.50–2.00	100	toluene	1000	[44]
Pt/SiO_2_	flame spray pyrolysis	0.21	100	benzene	100	[45]
Pd/TiO_2_	impregnation + reduction	1.00	500	formaldehyde	10	[46]
Pd/TiO_2_	deposition-precipitation + reduction	1.00	500	formaldehyde	10	[46]
Au/TiO_2_	deposition-precipitation	1.00	200	propene	1000	[47]
Ag/TiO_2_	impregnation	8.00	60	formaldehyde	110	[48]
Ag/CeO_2_/SiO_2_	impregnation	5.00	145	formaldehyde	18,000–22,000	[49]
